# The association between carbon and nitrogen stable isotope ratios of human hair and hypertension

**DOI:** 10.1186/s40885-022-00228-z

**Published:** 2023-02-01

**Authors:** Song Vogue Ahn, Jong-Ku Park

**Affiliations:** 1 Department of Health Convergence, Ewha Womans University, Seoul, Republic of Korea; 2 Department of Preventive Medicine, Yonsei University Wonju College of Medicine, Wonju, Republic of Korea

**Keywords:** Isotopes, Nitrogen, Carbon, Blood pressure, Hypertension

## Abstract

**Background:**

The relationship between stable isotope ratios and dietary protein sources has been reported. However, few studies have examined the effect of stable isotope ratios on metabolic risk in humans. We investigated whether the stable isotope ratios of carbon and nitrogen in human hair are associated with blood pressure and hypertension.

**Methods:**

We conducted a cross-sectional study of 392 subjects (228 men and 164 women). Hair samples of the subjects were used for the measurement of stable isotope ratios of carbon (δ^13^C) and nitrogen (δ^15^N).

**Results:**

The δ^13^C and δ^15^N values showed positive correlations with diastolic blood pressure in the subjects without antihypertensive medication. In the subjects without antihypertensive medication, the multivariable-adjusted odds ratio (95% confidence interval) for hypertension was 1.55 (1.04–2.30) per 1‰ increase in δ^15^N and 1.22 (0.86–1.73) per 1‰ increase in δ^13^C, respectively. However, in the subjects with antihypertensive medication, neither δ^13^C nor δ^15^N values showed a significant association with hypertension.

**Conclusions:**

The stable isotopic ratio of nitrogen in scalp hair is independently associated with hypertension in subjects without antihypertensive medication. The hair δ^15^N value might be used as a surrogate marker to screen a high-risk population for hypertension.

## Background

Hypertension is a major public health issue and is one of the most significant risk factors for cardiovascular disease, cerebrovascular accidents, and chronic kidney disease [1–4]. It has been found that prevention and control of hypertension could substantially reduce the risk for cardiovascular disease [5], and dietary and lifestyle factors play an important role in the development and management of hypertension [6].

In ecological and archeology investigations, stable isotope ratios have been utilized to provide quantitative information on food intake in animal and human bodies and the cycle of materials [7]. As animal and human body proteins reflect their dietary history, analysis of stable isotopes could determine the sources of foods and materials [8]. Carbon (^13^C/^12^C, δ^13^C) and nitrogen (^15^N/^14^N, δ^15^N) stable isotope ratios have lately been suggested as possible indicators for food intake and nutritional health [9–11]. It has been reported that those who consume more animal protein have higher δ^13^C and δ^15^N levels in scalp hair [11–14]. Although diet is a significant risk factor for hypertension and metabolic diseases [6, 15, 16], little is known regarding the relationship between stable isotope ratios and metabolic risk in humans. Results of recent studies have suggested the possible association of nitrogen stable isotope ratios with metabolic syndrome and serum leptin levels [17, 18].

Biomarkers may be beneficial in detecting and predicting the risk of chronic illnesses including hypertension, diabetes, and metabolic syndrome [19–21]. Despite the high prevalence of hypertension and its potential complications, the ability of carbon and nitrogen stable isotope ratios to function as biomarkers of high blood pressure is poorly understood. Thus, we investigated whether the ratios of carbon and nitrogen stable isotopes in human hair are associated with blood pressure and hypertension.

## Methods

### Study subjects

We conducted a cross-sectional study of stable isotopic analysis with 399 subjects (233 men and 166 women), which is an ancillary study of the community-based cohort study [17, 19]. Hair samples of study subjects were collected for the measurement of carbon and nitrogen stable isotope ratios. We excluded seven subjects with incomplete data and then included 392 subjects (228 men and 164 women) for the data analyses. The study protocol was approved by the Institutional Review Board of Yonsei University Wonju College of Medicine and was in compliance with the Declaration of Helsinki. Written informed consent was obtained from each study subject.

### Data collection

Medical and family history, lifestyle factors, dietary intake, and physical examinations were all collected using comprehensive questionnaires and completed according to standardized procedures [19]. Subjects’ blood pressures were measured after they had rested for at least 5 minutes in a quiet environment using a standard sphygmomanometer. An appropriate-sized cuff was wrapped around the upper right arm at heart level while subjects were sitting. Two measurements were taken with at least 5-minute intervals between them, and the mean of the two measurements of blood pressure was used for the analysis. Based on the 2018 Korean Society of Hypertension guidelines, hypertension was defined as systolic blood pressure ≥ 140 mmHg or diastolic blood pressure ≥ 90 mmHg [22]. Weights, heights, and waist circumferences were measured while subjects wore light indoor attire with no shoes. Smoking status, alcohol consumption, physical activity, and exercise were assessed based on self-report. A semiquantitative food frequency questionnaire designed for Korean adults was used to examine dietary intake [23], and nutrient consumption was estimated using the nutrient database. After fasting for 12 hours, subjects’ venous blood samples were taken. Fasting blood glucose was measured using a glucose oxidase-based assay, and the blood levels of high-density lipoprotein (HDL) cholesterol and triglyceride were determined using enzyme methods (ADVIA 1800; Siemens Healthcare Diagnostics, Tarrytown, NY, USA). Insulin resistance was estimated using the homeostasis model assessment of insulin resistance (HOMA-IR) method with the following formula: fasting insulin (μIU/mL) × fasting blood glucose (mg/dL) / 405 [24].

### Stable isotopic analysis

Hair samples were taken by being cut from the subject’s crown and as close to the scalp as feasible. A standard process was used to prepare the hair samples [7, 25]. Hairs were cleaned twice by soaking in a 2:1 mixture of methanol and chloroform for 30 minutes to remove lipids and hair product residues, then rinsed in distilled water for 15 minutes. Hair samples were wrapped in aluminum foil and cut into 15-mm sections, then vacuum-dried overnight to eliminate moisture. Carbon and nitrogen isotopes were analyzed using an isotope-ratio mass spectrometer (GV IsoPrime, Manchester, UK) connected to an elemental analyzer (EuroEA3000 series; Eurovector, Milano, Italy) at the Korea Basic Science Institute. The Dumas principle was used to operate the elemental analyzer, which involves dynamic flash combustion followed by gas chromatography column separation of the gaseous species produced [26]. Isotopic ratios are expressed in delta (δ) notation in parts per thousand (‰) relative to the accepted international standards: Vienna Pee Dee Belemnite for carbon isotopes and atmospheric air for nitrogen isotopes. The ratio is expressed as δ (‰) = [(R_x_ / R_s_) − 1] × 1000, where R_x_ is the ^13^C/^12^C or ^15^N/^14^N isotopic ratio of the sample, and R_s_ is the ^13^C/^12^C or ^15^N/^14^N isotopic ratio of the standard. The analytical precision was ±0.2‰ for δ^13^C and ± 0.3‰ for δ^15^N, respectively.

### Statistical analysis

We examined the distribution and normality of data and performed one-way analyses of variance, Kruskal-Wallis tests, or chi-square tests to compare differences in the profiles of study subjects. Pearson’s or Spearman’s correlation coefficients were calculated to test the correlation between δ^13^C or δ^15^N values and other variables. We used multivariable logistic regression models to evaluate the independent association of δ^13^C or δ^15^N values with hypertension. Five logistic regression models were used, each with a different degree of confounder adjustment. In model 1, we adjusted for age and sex. In model 2, we additionally adjusted for total energy intake and dietary sodium intake. In model 3, we additionally adjusted for fasting blood glucose, HDL cholesterol, and triglyceride. In model 4, we additionally adjusted for smoking status, alcohol consumption, and regular exercise. In model 5, we additionally adjusted for waist circumference. Further, we assessed the association of δ^13^C or δ^15^N values with hypertension stratified by antihypertensive medication. SAS ver. 9.4 (SAS Institute Inc., Cary, NC, USA) was used for all statistical analyses. All comparisons were considered statistically significant if the *P*-value was less than 0.05.

## Results

A total of 175 subjects had hypertension in 392 study subjects. Of the 175 hypertensive subjects, 45.1% were taking antihypertensive medications. Table 1 shows the profiles of the study subjects. Age, body mass index, waist circumference, fasting blood glucose, HOMA-IR, triglyceride, and the proportion of lipid-lowering medication were the highest, and total cholesterol and HDL cholesterol were the lowest in the hypertensive subjects with antihypertensive medication.

**Table 1:** Characteristics of study subjects

Characteristic	Non-hypertensive subjects	Hypertensive subjects with antihypertensive medication	Hypertensive subjects without antihypertensive medication	*P*-value^a)^
Number	217	79	96	–
Age (yr)	60.7 ± 8.6	63.6 ± 7.3	62.1 ± 7.6	0.015
Male sex	120 (55.3)	51 (64.6)	57 (59.4)	0.018
δ^13^C, ‰ vs. PDB	−20.4 ± 0.9	−20.6 ± 0.9	−20.3 ± 0.8	0.095
δ^15^N, ‰ vs. air	11.5 ± 0.8	11.6 ± 0.8	11.7 ± 0.8	0.075
Body mass index (kg/m^2^)	23.5 ± 3.1	25.2 ± 3.4	24.4 ± 3.2	< 0.001
Waist circumference (cm)	82.3 ± 8.4	87.1 ± 8.8	84.2 ± 7.7	< 0.001
Systolic blood pressure (mmHg)	119.7 ± 9.6	132.0 ± 15.4	144.1 ± 9.6	< 0.001
Diastolic blood pressure (mmHg)	74.6 ± 7.3	79.9 ± 9.8	88.8 ± 9.0	< 0.001
Fasting glucose (mg/dL)	97.7 ± 17.3	103.4 ± 16.2	97.4 ± 11.8	0.015
HOMA-IR (unit)	1.6 (1.2–2.1)	1.9 (1.5–3.0)	1.7 (1.3–2.1)	0.001
Total cholesterol (mg/dL)	184.3 ± 31.7	175.2 ± 31.5	188.3 ± 30.1	0.020
Triglyceride (mg/dL)	111 (78–150)	138 (109–217)	126 (95–167)	< 0.001
HDL cholesterol (mg/dL)	54.2 ± 11.3	50.4 ± 12.1	54.2 ± 14.1	0.046
Lipid-lowering medication	3 (1.38)	13 (16.5)	0	< 0.001
Total energy intake (kcal/day)	1671.1 ± 532.1	1658.3 ± 411.0	1701.4 ± 556.9	0.843
Sodium intake (mg/day)	2965.5 ± 1734.6	3070.6 ± 1984.5	3260.1 ± 1913.9	0.452
Current smoker	36 (16.6)	10 (12.7)	16 (16.7)	0.690
Alcohol drinker	105 (48.4)	49 (62.0)	54 (56.3)	0.089
Regular exercise	58 (26.7)	25 (31.6)	32 (33.3)	0.437

Data are presented as mean ± standard deviation, number (%), or median (25th–75th percentile)

*PDB* Pee Dee Belemnite, *HOMA-IR* Homeostasis model assessment of insulin resistance, *HDL* High-density lipoprotein

^a)^*P*-value from Kruskal-Wallis test

In all subjects, the δ^13^C values showed positive correlations with diastolic blood pressure (r = 0.119, *P* = 0.018), waist circumference (r = 0.150, *P* = 0.003), and total energy intake (r = 0.145, *P* = 0.004), and showed negative correlation with age (r = − 0.226, *P* < 0.001). The δ^15^N values showed positive correlations with diastolic blood pressure (r = 0.117, *P* = 0.021), body mass index (r = 0.207, *P* < 0.001), waist circumference (r = 0.316, P < 0.001), fasting glucose (r = 0.122, *P* = 0.015), and total energy intake (r = 0.168, P < 0.001), and showed negative correlations with age (r = − 0.123, P = 0.015) and HDL cholesterol (r = − 0.120, *P* = 0.018) in all subjects. In the subjects without antihypertensive medication, the δ^13^C values showed positive correlations with diastolic blood pressure (r = 0.157, *P* = 0.005), waist circumference (r = 0.165, *P* = 0.004), fasting glucose (r = 0.123, *P* = 0.030), and total energy intake (r = 0.141, *P* = 0.013), and showed negative correlation with age (r = − 0.231, *P* < 0.001). The δ^15^N values showed positive correlations with systolic blood pressure (r = 0.113, *P* = 0.045), diastolic blood pressure (r = 0.179, *P* = 0.001), body mass index (r = 0.174, *P* = 0.002), waist circumference (r = 0.295, *P* < 0.001), fasting glucose (r = 0.135, *P* = 0.017), and total energy intake (r = 0.191, P < 0.001), and showed negative correlation with age (r = − 0.166, *P* = 0.003) in the subjects without antihypertensive medication (Table 2).

**Table 2:** Correlations between δ^13^C, δ^15^N values and other variables

Variable	Correlation coefficients (*P*-value)	
	δ^13^C, ‰ vs. PDB	δ^15^N, ‰ vs. air
All subjects (*n* = 392)		
Systolic blood pressure (mmHg)	0.013 (0.799)	0.059 (0.247)
Diastolic blood pressure (mmHg)	0.119 (0.018)	0.117 (0.021)
Age (yr)	−0.226 (< 0.001)	− 0.123 (0.015)
Body mass index (kg/m^2^)	0.063 (0.210)	0.207 (< 0.001)
Waist circumference (cm)	0.150 (0.003)	0.316 (< 0.001)
Fasting glucose (mg/dL)	0.037 (0.462)	0.122 (0.015)
HOMA-IR (unit)	−0.033^a)^ (0.519)	0.072^a)^ (0.152)
Total cholesterol (mg/dL)	0.021 (0.681)	0.016 (0.746)
Triglyceride (mg/dL)	0.053^a)^ (0.293)	0.031^a)^ (0.536)
HDL cholesterol (mg/dL)	0.045 (0.378)	−0.120 (0.018)
Total energy intake (kcal/day)	0.145 (0.004)	0.168 (< 0.001)
Sodium intake (mg/day)	0.003 (0.959)	−0.024 (0.642)
Subjects without antihypertensive medication (*n* = 313)		
Systolic blood pressure (mmHg)	0.042 (0.462)	0.113 (0.045)
Diastolic blood pressure (mmHg)	0.157 (0.005)	0.179 (0.001)
Age (yr)	−0.231 (< 0.001)	− 0.166 (0.003)
Body mass index (kg/m^2^)	0.065 (0.252)	0.174 (0.002)
Waist circumference (cm)	0.165 (0.004)	0.295 (< 0.001)
Fasting glucose (mg/dL)	0.123 (0.030)	0.135 (0.017)
HOMA-IR (unit)	−0.022^a)^ (0.700)	0.080^a)^ (0.160)
Total cholesterol (mg/dL)	0.004 (0.937)	0.070 (0.220)
Triglyceride (mg/dL)	0.114^a)^ (0.043)	0.052^a)^ (0.360)
HDL cholesterol (mg/dL)	0.002 (0.966)	−0.096 (0.090)
Total energy intake (kcal/day)	0.141 (0.013)	0.191 (< 0.001)
Sodium intake (mg/day)	−0.065 (0.266)	−0.061 (0.298)

Data are presented as Pearson correlation coefficients (*P*-value)

^a)^ Spearman rank correlation coefficients (*P*-value)

*PDB* Pee Dee Belemnite, *HOMA-IR* Homeostasis model assessment of insulin resistance, *HDL* High-density lipoprotein

**Fig. 1:**
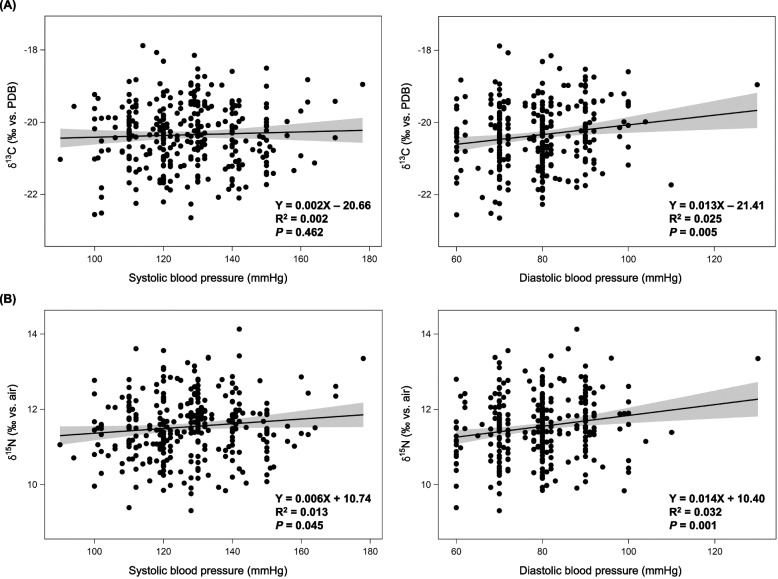
Relation of δ^13^C and δ^15^N values to systolic and diastolic blood pressures in 313 subjects without antihypertensive medication. **A** δ^13^C. **B** δ^15^N. The scatter plots are shown with regression lines (black solid lines) and 95% confidence intervals (gray areas). PDB, Pee Dee Belemnite

**Fig. 2:**
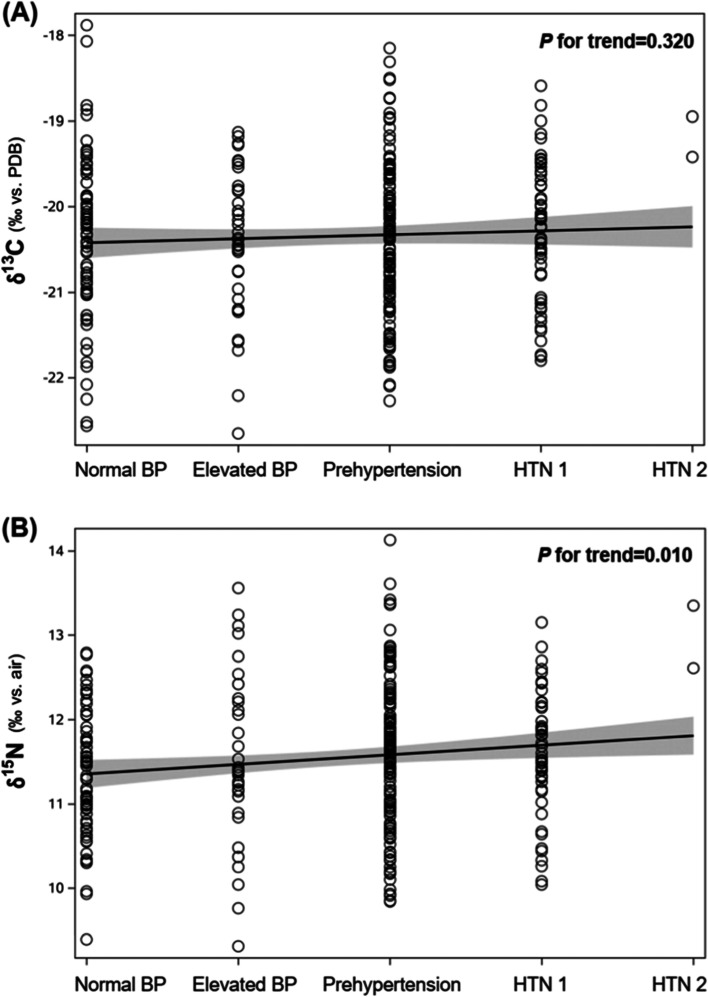
Relation of δ^13^C and δ^15^N values to the Korean Society of Hypertension classification of blood pressure in 313 subjects without antihypertensive medication. **A** δ^13^C. **B** δ^15^N. The scatter plots are shown with regression lines (black solid lines) and 95% confidence intervals (gray areas). Normal blood pressure (BP), systolic BP (SBP) < 120 mmHg and diastolic BP (DBP) < 80 mmHg; elevated BP, SBP 120–129 mmHg and DBP < 80 mmHg; prehypertension, SBP 130–139 mmHg or DBP 80–89 mmHg; hypertension grade 1 (HTN 1), SBP 140–159 mmHg or DBP 90–99 mmHg; hypertension grade 2 (HTN 2), SBP ≥160 mmHg or DBP ≥100 mmHg. PDB, Pee Dee Belemnite

The associations between δ^13^C or δ^15^N values and systolic and diastolic blood pressures were visualized by regression analyses (Fig. 1). The δ^15^N values showed positive correlations with both systolic blood pressure (R^2^ = 0.013, *P* = 0.045) and diastolic blood pressure (R^2^ = 0.032, *P* = 0.001). The δ^13^C values showed significant correlations with diastolic blood pressure (R^2^ = 0.025, *P* = 0.005). Furthermore, δ^15^N values progressively increased with the blood pressure classification of the Korean Society of Hypertension in the subjects without antihypertensive medication (P for trend =0.010) (Fig. 2) [22].

Table 3 shows odds ratios (ORs) for hypertension according to δ^13^C and δ^15^N tertiles in the subjects without antihypertensive medication. The ORs for hypertension comparing subjects in the highest to those in the lowest tertile of δ^13^C and δ^15^N values were 1.12 (95% confidence interval [CI], 0.54–2.29; P for trend = 0.769) and 2.19 (95% CI, 1.03–4.65; P for trend = 0.040), respectively, in multivariable models, adjusted for age, sex, total energy intake, dietary sodium intake, fasting blood glucose, triglyceride, HDL cholesterol, smoking, alcohol consumption, regular exercise, and waist circumference.

**Table 3:** Odds ratios for hypertension according to δ^13^C, δ^15^N tertiles in 313 subjects without antihypertensive medication

Variable	Odds ratio (95% confidence interval)				
	Terrtile 1	Terrtile 2	Terrtile 3	P for trend	per 1‰ increase
δ^13^C, ‰ vs. PDB	<−20.71	−20.71 to −19.99	≥ − 19.98	–	–
Hypertension	40 (29.4)	44 (33.6)	41 (32.8)	–	–
Model 1	1.00	0.98 (0.53–1.84)	1.21 (0.61–2.38)	0.583	1.24 (0.89–1.73)
Model 2	1.00	0.98 (0.52–1.83)	1.20 (0.61–2.36)	0.602	1.24 (0.89–1.73)
Model 3	1.00	0.94 (0.50–1.77)	1.17 (0.59–2.34)	0.643	1.23 (0.88–1.73)
Model 4	1.00	0.94 (0.49–1.77)	1.10 (0.54–2.26)	0.788	1.22 (0.86–1.72)
Model 5	1.00	0.93 (0.49–1.76)	1.12 (0.54–2.29)	0.769	1.22 (0.86–1.73)
δ^15^N, ‰ vs. air	< 11.18	11.18 to 11.85	≥11.86	–	–
Hypertension	35 (27.6)	43 (33.3)	47 (34.6)	–	–
Model 1	1.00	1.55 (0.81–2.95)	2.31 (1.13–4.74)	0.022	1.57 (1.08–2.28)
Model 2	1.00	1.57 (0.82–3.00)	2.32 (1.13–4.76)	0.021	1.56 (1.07–2.28)
Model 3	1.00	1.61 (0.84–3.11)	2.33 (1.12–4.85)	0.024	1.61 (1.10–2.37)
Model 4	1.00	1.54 (0.79–3.01)	2.28 (1.09–4.80)	0.029	1.58 (1.07–2.33)
Model 5	1.00	1.50 (0.77–2.94)	2.19 (1.03–4.65)	0.040	1.55 (1.04–2.30)

Model 1, adjusted for age, sex; Model 2, model 1 + additionally adjusted for total energy intake, dietary sodium intake; Model 3, model 2 + additionally adjusted for fasting glucose, triglyceride, high-density lipoprotein cholesterol; Model 4, model 3 + additionally adjusted for smoking, alcohol consumption, regular exercise; Model 5, model 4 + additionally adjusted for waist circumference; PDB, Pee Dee Belemnite

**Fig. 3:**
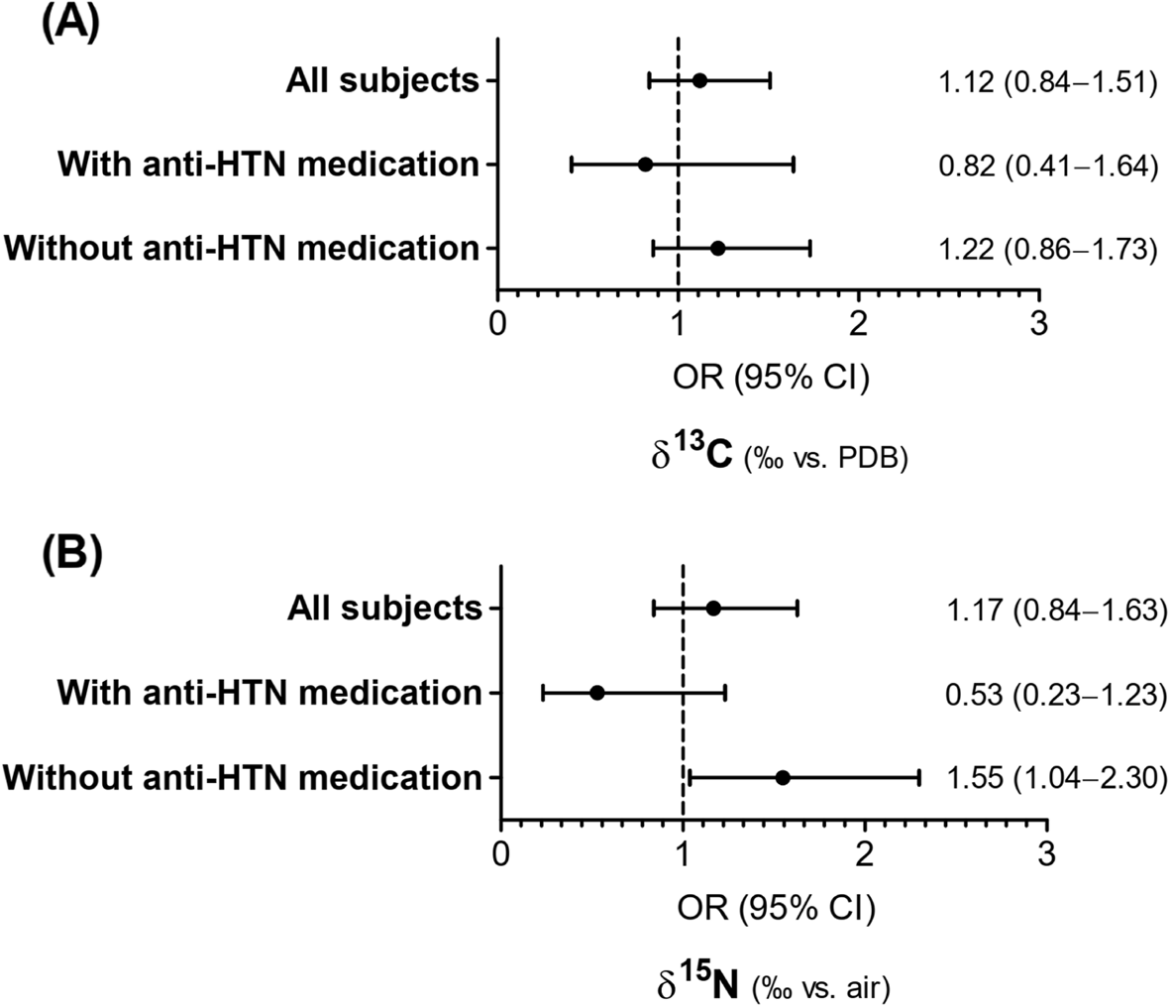
Odds ratios (ORs) for hypertension according to δ^13^C and δ^15^N values by antihypertensive (anti-HTN) medication. **A** δ^13^C. **B** δ^15^N. ORs were calculated after adjusting for age, sex, total energy intake, dietary sodium intake, fasting serum glucose, triglyceride, high-density lipoprotein cholesterol, smoking, alcohol consumption, regular exercise, and waist circumference. CI, confidence interval; PDB, Pee Dee Belemnite

In all subjects, the corresponding OR (95% CI) per 1‰ increase in δ^15^N for hypertension was 1.17 (0.84–1.63) after adjusting for age, sex, total energy intake, dietary sodium intake, fasting blood glucose, triglyceride, HDL cholesterol, smoking, alcohol consumption, regular exercise, and waist circumference. When we divided subjects by antihypertensive medication, the positive association between a 1‰ increase in δ^15^N and hypertension was significant in subjects without antihypertensive medication (OR, 1.55; 95% CI, 1.04–2.30), whereas the association between a 1‰ increase in δ^15^N and hypertension was not significant in subjects with antihypertensive medication (OR, 0.53; 95% CI, 0.23–1.23). A 1‰ increase in δ^13^C was not associated with hypertension in all subjects and any subgroup of antihypertensive medication (Fig. 3).

## Discussion

The nitrogen, but not carbon, stable isotopic ratio is independently associated with hypertension in the subjects without antihypertensive medication. Higher δ^15^N values of hair were associated with an increased risk of hypertension. However, in the subjects with antihypertensive medication, neither δ^13^C nor δ^15^N values showed a significant association with hypertension. This is the first research to explore the potential clinical implications of δ^15^N values in relation to hypertension.

Stable isotopic analysis of hair has been known as a noninvasive technology that provides a long-term reflection of food consumption [10]. Previous studies have found a positive correlation between animal protein intake and δ^13^C and δ^15^N values [13, 27]. Nitrogen stable isotope ratios are known to be nutritional indicators for dietary meat consumption [7, 13, 28], particularly red and processed meat, which might explain the association between δ^15^N values and hypertension. High amounts of meat intake have been associated with a higher risk of hypertension in previous studies [6, 29], which might have contributed to the link between δ^15^N and hypertension. A UK research reported that those who consumed more animal protein showed higher δ^15^N values than vegans, suggesting that nitrogen stable isotope ratios might be utilized as a nutritional biomarker for dietary meat consumption [7]. Higher nitrogen stable isotope ratios in the human body are attributed to the kinetic fractionation of nitrogen stable isotopes during the deamination and transamination of amino acids, which results in increased excretion of lighter nitrogen isotopes and enrichment of ^15^N in the body [12].

δ^15^N values might be also an indicator of other metabolic risk factors, such as heavy metals or persistent organic pollutants, which are known to be associated with hypertension [30–32]. Bioaccumulation trends in the marine food chain are similar for nitrogen stable isotopes, heavy metals, and persistent organic contaminants [33–35]. The nitrogen stable isotope ratio is reported to be correlated with mercury exposure [36], which could be associated with high blood pressure and hypertension [37]. Given that δ^15^N values were related to fish consumption, the δ^15^N values associated with fish consumption might be linked to heavy metals or persistent organic pollutants, perhaps contributing to the positive correlation of δ^15^N values with hypertension [38, 39]. More research is needed to understand the link between δ^15^N values and blood pressure.

In the present study, the association of δ^15^N values with hypertension was only found in the subjects not taking antihypertensive drugs, and no such association was observed in the subjects taking antihypertensive drugs. The use of antihypertensive drugs, which lower blood pressure, might alter the association between blood pressure levels and δ^15^N values. In addition, our findings might be attributed to the difference in lipid profiles between the subjects with and without antihypertensive medication. Dyslipidemia has been known to be associated with hypertension [40], and previous studies have reported that blood lipid profiles including total cholesterol, HDL cholesterol, LDL cholesterol, and triglyceride were higher in patients with uncontrolled hypertension compared to those with controlled hypertension [41–43]. Those findings seem to be consistent with our results that blood levels of total cholesterol and HDL cholesterol were higher in the subjects without antihypertensive medication than in those with antihypertensive medication. Additionally, the proportion of lipid-lowering medication was higher in the subjects with antihypertensive medication than in those without antihypertensive medication.

Our findings showed that δ^13^C values of hair were not associated with hypertension. The proportion of C3 plants (e.g., rice, wheat, fruits, and vegetables) and C4 plants (e.g., cane and maize) in a person’s diet determines the carbon stable isotope ratios in the human body [12, 44], δ^13^C values might be employed as a biomarker for sugar-sweetened drinks containing C4 sugars [12, 44]. Dietary carbohydrate intake could be associated with the risk of hypertension [45], but δ^13^C values have been reported to show no significant correlation with carbohydrate intake in Korean diets [17]. The δ^13^C values also have been reported to be inversely associated with dietary fish intake and diabetes [28, 46]. The complicated and contradictory relationships of δ^13^C with various factors might explain the lack of association between δ^13^C and hypertension.

There are several limitations to this study that should be considered. First, the direction of a causal relationship between nitrogen stable isotope ratios and hypertension could not be established because this study has a cross-sectional design. Longitudinal studies may be necessary to clarify the direction of causation. Second, because this study only included elderly Koreans, the findings may be limited in their generalizability. Third, measurement error may exist, particularly due to random within-person variation although lifestyle factors and dietary intake were measured according to standardized procedures.

## Conclusions

The stable isotopic ratio of nitrogen in scalp hair is independently associated with hypertension in subjects without antihypertensive medication. The hair δ15N value might be used as a surrogate marker to screen a high-risk population for hypertension. Further research is needed to fully comprehend the mechanism behind the association between stable isotope ratios and blood pressure.

## Data Availability

The datasets used and/or analyzed during the current study are available from the corresponding author on reasonable request.

## Abbreviations

Anti-HTN: Antihypertensive
BP: Blood pressure
CI: Confidence interval
DBP: Diastolic blood pressure
HDL: High-density lipoprotein
HOMA-IR: Homeostasis model assessment of insulin resistance
HTN 1: Hypertension grade 1
HTN 2: Hypertension grade 2
KSH: Korean Society of Hypertension
OR: Odds ratio
PDB: Pee Dee Belemnite
SBP: Systolic blood pressure
